# Impact of encorafenib on survival of patients with BRAF^V600E^-mutant metastatic colorectal cancer in a real-world setting

**DOI:** 10.1007/s00432-023-05141-y

**Published:** 2023-07-19

**Authors:** M. Zurloh, M. Goetz, T. Herold, J. Treckmann, P. Markus, B. Schumacher, D. Albers, A. Rink, V. Rosery, G. Zaun, K. Kostbade, M. Pogorzelski, S. Ting, H. Schmidt, R. Stiens, M. Wiesweg, M. Schuler, Stefan Kasper, I. Virchow

**Affiliations:** 1 Department of Medical Oncology, West German Cancer Center, University Hospital Essen, Hufelandstr. 55, 45147 Essen, Germany; 2 West German Cancer Center, Institute of Pathology Essen, University Hospital Essen, Essen, Germany; 3 West German Cancer Center, Department of General, Visceral and Transplant Surgery, University Hospital Essen, Essen, Germany; 4 Department of General Surgery and Traumatology, Elisabeth Hospital, Essen, Germany; 5 Department of Gastroenterology, Elisabeth Hospital, Essen, Germany; 6 Institute of Pathology Nordhessen, Kassel, Germany; 7 Department of Gastroenterology, Hepatology and Transplant Medicine, University Hospital Essen, Essen, Germany; 8 German Cancer Consortium (DKTK), Partner Site University Hospital Essen, Essen, Germany; 9 Medical Faculty, University Duisburg-Essen, Essen, Germany

**Keywords:** Metastatic colorectal cancer, BRAFV600E, Chemotherapy, Encorafenib

## Abstract

**Purpose:**

Patients with *BRAF*^*V600E*^-mutant metastatic colorectal cancer (mCRC) have a dismal prognosis. The best strategies in these patients remain elusive. Against this background, we report the clinical course of patients with *BRAF*^*V600E*^-mutant mCRC to retrieve the best treatment strategy.

**Patients and methods:**

Clinico-pathological data were extracted from the electronic health records. Kaplan–Meier method was used to estimate overall (OS) and progression-free survival (PFS). Objective response rate (ORR) was assessed according to RECIST 1.1.

**Results:**

In total, 51 patients were enrolled. FOLFOXIRI was administered to 12 patients; 29 patients received FOLFOX or FOLFIRI as first-line treatment. Median OS was 17.6 months. Median PFS with FOLFOXIRI (13.0 months) was significantly prolonged (HR 0.325) as compared to FOLFOX/FOLFIRI (4.3 months). However, this failed to translate into an OS benefit (*p* = 0.433). Interestingly, addition of a monoclonal antibody to chemotherapy associated with superior OS (HR 0.523). A total of 64.7% patients received further-line therapy, which included a BRAF inhibitor in 17 patients. Targeted therapy associated with very favourable OS (25.1 months).

**Conclusion:**

Patients with BRAF^V600E^-mutated mCRC benefit from the addition of an antibody to first-line chemotherapy. Further-line treatment including a BRAF inhibitor has a dramatic impact on survival.

**Supplementary Information:**

The online version contains supplementary material available at 10.1007/s00432-023-05141-y.

## Introduction

Colorectal cancer (CRC) is the third most prevalent cancer and the second leading cause of cancer related death in western countries (Bray et al. 2018). Resection of the primary tumour with or without systemic adjuvant chemotherapy is a curative option in patients with local or locally advanced disease. However, up to 50% of patients initially present with distant metastases or develop distant relapse. Systemic chemotherapy is the primary choice of treatment for patients not amenable to curative surgery or metastasectomy. A comprehensive molecular profiling including mutational analysis of *KRAS* (Kirsten rat sarcoma virus), *NRAS* (neuroblastoma RAS viral oncogene homolog), *BRAF* (RAS-associated factor B) and dMMR/MSI (deficient mismatch repair/microsatellite instability) is recommended before initiation of systemic palliative treatment (Benson et al. 2021). In addition, primary tumour localization (right sided vs left sided) has an impact on prognosis and efficacy of several drugs and should be considered before the initiation of first-line systemic treatment (Arnold et al. 2017). Mutations of the *KRAS* or *NRAS* oncogene are detected in up to 55% of all mCRCs and are strong negative predictors for the efficacy of monoclonal antibodies targeting the epidermal growth factor receptor (EGFR) (Stintzing et al. 2017; Venook et al. 2017). Thus, patients with *KRAS-* or *NRAS*-mutant tumours should be treated with a chemotherapy doublet (FOLFOX or FOLFIRI) or triplet (FOLFOXIRI) in combination with the anti-angiogenic antibody bevacizumab targeting the vascular endothelial growth factor A (VEGFA) in first line. Patients with mCRC without *KRAS* or *NRAS* mutations (all RAS wildtype) should be treated with a chemotherapy doublet in combination with an EGFR antibody (cetuximab or panitumumab) rather than with bevacizumab in first line. However, efficacy of EGFR antibodies seems to be restricted to patients whose primary tumour is located on the left site of the colon, whilst patients harbouring a tumour situated proximal from the left splenic flexure should rather receive bevacizumab in first line (Arnold et al. 2017).

Mutations of the *BRAF* oncogene, primarily BRAF^V600E^, are detected in up to 10% of patients with colorectal cancer (Fransen et al. 2004; Souglakos et al. 2009; Roth et al. 2010; Yokota et al. 2011; Clancy et al. 2013; Sinicrope et al. 2015). Colorectal cancers with *BRAF*^*V600E*^ mutations are clinically different from tumours without BRAF mutations and also from tumours harbouring non-V600 mutations (Jones et al. 2017). BRAF^V600E^-mutant tumours are more frequently observed in female patients and situated right-sided (Loupakis et al. 2016). Additionally, these tumours are associated with a poor histopathological grading, MSI^high^/dMMR status, larger primary tumour size, higher rate of nodal metastases and peritoneal carcinomatosis. Patients with *BRAF*^*V600E*^*-*mutant metastatic CRC have a dismal prognosis with a reduced response to chemotherapy and dramatically reduced overall survival time of only 1 year in clinical trials (Cremolini et al. 2015; Modest et al. 2016; Stintzing et al. 2017).

The best treatment strategy in this poor prognostic subgroup of patients with BRAF^V600E^-mutant mCRC remains elusive. Current clinical guidelines recommend a triplet chemotherapy with FOLFOXIRI in combination with bevacizumab as preferred first-line chemotherapy in fit patients (Van Cutsem et al. 2016). However, these recommendations were based on a small retrospective subgroup analysis of 28 patients treated within the TRIBE study (Cremolini et al. 2015). In this study, the triplet chemotherapy with FOLFOXIRI plus bevacizumab was superior to the doublet with FOLFIRI plus bevacizumab. Recently, pooled analyses of 5 clinical trials which tested the triplet FOLFOXIRI plus bevacizumab versus a doublet chemotherapy with FOLFOX or FOLFIRI plus bevacizumab showed comparable median overall survival with the triplet (13.6 months) or the doublet chemotherapy (14.5 months) in at least 115 patients with BRAF^V600E^-mutated metastatic CRC (Cremolini et al. 2020). In a real-world setting, a substantial number of patients do not qualify for the triplet chemotherapy with FOLFOXIRI due to age, reduced performance status or comorbidities. The efficacy of EGFR directed antibodies (cetuximab, panitumumab) in BRAF^V600E^-mutated mCRC has been discussed controversially (Pietrantonio et al. 2015; Rowland et al. 2015). Retrospective post-hoc meta-analyses of large clinical trials did not show conclusive results. However, the recently published FIRE4.5 study demonstrated that the triplet chemotherapy with a modified FOLFOXIRI plus the EGFR antibody cetuximab was inferior to FOLFOXIRI plus bevacizumab in first-line setting (Stintzing et al. 2021). Interestingly, after failure of cytotoxic chemotherapy, the combination of the BRAF inhibitor encorafenib and the monoclonal EGFR antibody cetuximab is effective in patients with BRAF^V600E^-mutant mCRC. In the randomized phase III BEACON trial, this combination was superior to a standard systemic chemotherapy and became the new standard for previously treated patients (Kopetz et al. 2019).

In the above-reported prospective clinical trials, only patients in good performance status without significant comorbidities were enrolled. The results of these trials are thus not fully applicable to patients treated in a real-world setting. Treatment strategies in patients not fulfilling generic study inclusion criteria have to be defined. Against this background, we report the clinical course of a cohort of 51 patients with *BRAF*^*V600E*^-mutant mCRC who consulted our centre and received palliative treatment irrespective of predefined capability criteria such as performance status, age, comorbidities or laboratory values as required for clinical trial inclusion. The aim of this retrospective study is to define the best treatment strategy in this poor prognostic patient population. Further, we wanted to evaluate the impact of the introduction of BRAF inhibitor treatment in a real-world setting.

## Materials and methods

### Study design

Patients with histologically confirmed *BRAF*-mutant CRC diagnosed between 2008 and 2020 at the West German Cancer Center, Essen, Germany, were retrospectively identified and enrolled into this study. Patients were evaluable for analyses if they had a *BRAF* p.V600E mutation and were treated in the palliative setting (relapse or distant metastases) at our centre. Patients with atypical BRAF mutations and patients who were only diagnosed or had surgery at our centre but did not receive palliative treatment, or had no clinical follow-up data, were excluded (Suppl. Figure 1). Palliative treatment decision was made by the multidisciplinary tumour board (MTB), and chemotherapy regimen was selected by the treating oncologist based on the current clinical guidelines, performance status, comorbidities and patient wish. Follow-up data were routinely assessed and documented in the electronic health record (EHR). Clinical parameters and applied chemotherapy protocols including efficacy data were also retrieved from the EHR. Personal patient data were anonymized in the data base, and the data were analysed by a blinded researcher. The study was approved by the Ethics Committee of the Medical Faculty of the University Duisburg-Essen (Project No. 05-2882).

### Assessments

Tumour staging was performed according to the American Joint Committee on Cancer (AJCC)/International Union against Cancer (UICC) TNM classification (7^th^ Edition). Clinical staging was routinely based on computed tomography (CT) or magnetic resonance imaging (MRI) before start of palliative chemotherapy and subsequently every 8–12 weeks according to the institutional guidelines. Objective response rate (ORR) was evaluated according to the Response Evaluation Criteria in Solid Tumours 1.1 (RECIST 1.1). Patients were eligible for ORR assessment if they had a baseline radiological examination and at least one examination during the palliative chemotherapy. ORR was defined as the proportion of patients with complete or partial remission, the disease control rate (DCR) was defined as the proportion of patients with complete or partial remission or sustained disease stabilization at first radiological follow-up. Progression-free survival (PFS) was defined as time from start of chemotherapy to date of radiological or clinical progression or death. Overall survival was defined as time from start of palliative therapy to death. Patients were censored at the time of last visit at our centre, if time of death was not documented.

### Statistical analysis

All analyses were conducted using SPSS Statistics (V27, IBM, Armonk, NY, USA). Correlation analyses were performed using Pearson’s Chi-square test. Kaplan–Meier calculations with the log-rank test were used for analysis of OS and PFS. For follow-up time, the reverse Kaplan–Meier method was used. Univariate and multivariate analyses were performed by a Cox proportional-hazard model. Hazard ratio (HR) and 95% confidence intervals (CI) were indicated. Overall, *p* values ≤ 0.05 were regarded statistically significant.

## Results

### Patients’ characteristics

In total, 81 colorectal cancer patients with *BRAF* mutations were identified in our data base. For 23 of these patients, no clinical data were available and they were only diagnosed at our centre. Two of the 58 remaining patients had an atypical *BRAF* mutation (p.G469A and p.D594N) and were excluded from our analyses. Additional 5 patients were treated in curative intent and had no relapse or developed metastases till the data cut-off. The study population thus contained 51 patients with BRAF p.V600E-mutant mCRC, receiving palliative treatment at out centre. The median follow-up time was 68.4 months (range 2.7–99.3). Baseline characteristics are summarized in Table 1 and suppl. table 1. Median age at diagnosis was 61.9 years (range 40.5–88.4) and 62.2 years (range 41.4–88.4) at time of relapse; 54.9% patients were female, and 72.5% had a primary tumour located at the right side of the colon. Most patients had stage IV disease at time of diagnosis (72.5%), the remaining 14 patients (27.5%) developed metachronous metastases. The primary tumour was resected in 74.5% of all patients, and 62.3% of patients with initially non-metastatic disease received an adjuvant chemotherapy. The relapse-free survival (RFS) in those patients without synchronous metastases was 12 months (95% CI 7.6–16.4) (suppl. Figure 2).

**Table 1:** Patients’ characteristics

	*N*	%
Gender		
Male	23	45.1
Female	38	54.9
Age at diagnosis		
Median (range)	61.9 years (40.5–88.4)	
Age at metastases/relapse		
Median (range)	62.2 years (41.4–88.4)	
Tumour localization		
Left sided	12	23.5
Right sided	37	72.5
Unknown/both	2	3.9
M-stage		
Synchronous M1	37	72.5
Metachronous M1	14	27.5
Neoadjuvant therapy		
Yes	3	5.9
No	48	94.1
Resection of primary tumour		
Yes	38	74.5
No	13	25.5
Adjuvant CTX (only resected with metachronous metastases; *N *= 14)		
Yes	9	62.3
No	5	35.7
Type of adjuvant CTX		
FOLFOX/CAPOX	6	66.6
5-FU/Capecitabin	3	33.3

### Palliative first-line therapy

**Fig. 1:**
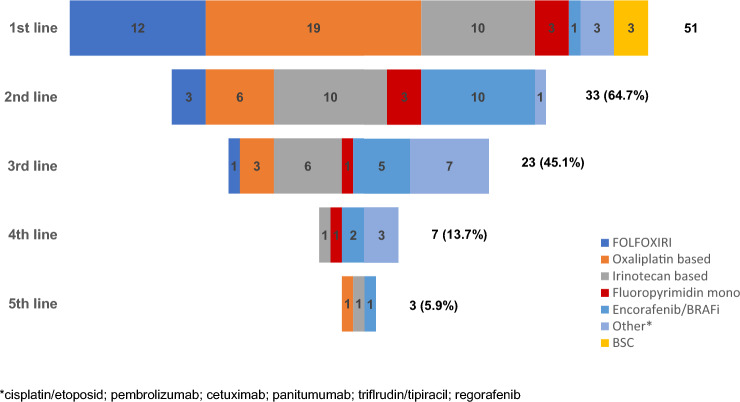
Funnel chart of palliative treatment regimen and conversion in first, second, third, fourth and fifth treatment line. *FOLFOXIRI* 5-fluorouracil, folinic acid, oxaliplatin, irinotecan, *BRAFi* BRAF inhibitor, *BSC* best supportive care

**Fig. 2:**
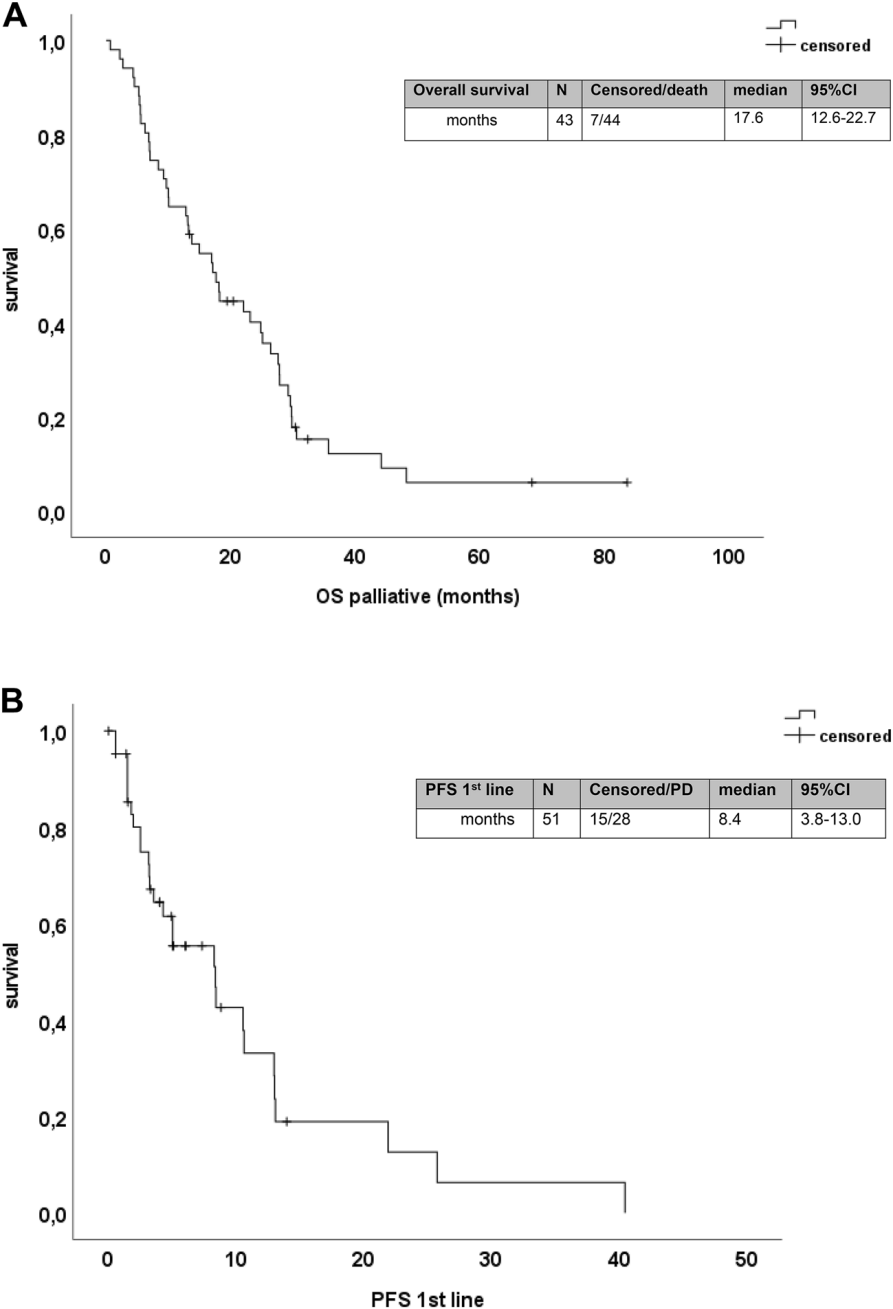
**a** Kaplan–Meier analysis for overall survival from start of palliative treatment. **b** Kaplan–Meier analysis for progression-free survival to first-line therapy

Palliative systemic treatment was administered to 48 patients (94.1%), whereas 3 (5.9%) patients only received best supportive care (BSC). Median number of therapy lines in the palliative setting was 2 (range 0–5); 33 (64.7%), 23 (45.1%), 7 (13.7%) and 3 (5.9%) of patients received second-, third-, fourth- or fifth-line therapy, respectively (Tables 2, 3, Fig. 1 and suppl. table 2). In total, 12 patients (23.5%) received triplet chemotherapy with FOLFOXIRI, 19 patients (37.3%) received oxaliplatin-based doublet chemotherapy, and 10 patients (19.6%) received irinotecan-based first-line therapy. A monoclonal antibody was added to first-line therapy in 31 patients (60.8%), 21 patients (41.2%) received bevacizumab, and 10 patients (19.6%) received an EGFR antibody (cetuximab or panitumumab). Objective response rate (ORR) according to RECIST 1.1 was evaluable for 39 patients. None of these patients had a complete remission (CR), 13 patients (33.3%) had a partial remission (PR), 10 patients (25.6%) had stable disease (SD), and 16 patients (41.0%) had a progressive disease (PD). Thus, the ORR was 33.3%, and the disease control rate (DCR) was 59.0% upon first-line therapy (Table 4). The median PFS of all enrolled patients (*N* = 51) was 8.4 months (95% CI 3.8–13.0), and the median OS from start of palliative treatment was 17.6 months (95% CI 12.6–22.7) (Fig. 2a, b).

**Table 2:** Palliative first-line therapy

	*N*	%
1st CTX received		
Yes	48	94.1
No (BSC)	3	5.9
Lines of therapy		
0	3	5.9
1	15	29.4
2	10	19.6
3	16	31.4
> 3	4	7.8
Median	2 (range 0–5)	
Triplet CTX (FOLFOXIRI)		
Yes	12	23.5
No	38	74.5
Unknown	1	2.0
First-line monoclonal antibody		
Bevacizumab	21	41.2
EGFR (Panitumumab/Cetuximab)	10	19.6
None	19	37.3
Unknown	1	2.0
First-line chemotherapy backbone		
Oxaliplatin-based (FOLFOX/CAPOX)	19	37.3
Irinotecan-based (FOLFIRI, Irinotecan)	10	19.6
FOLFOXIRI	12	23.5
Fluoropyrimidin mono	3	5.9
Other	2	3.9
Encorafenib	1	2.0
BSC	3	5.9
n.d.	1	2.0

**Table 3:** Systemic second-line therapy

	*N*	%
Second-line CTX received		
Yes	33	64.7
No (BSC)	18	35.3
Second-line monoclonal antibody (*N* = 33)		
Anti-angiogenic (Bev/Afli/Ram)	11	33.3
EGFR (Panitumumab/Cetuximab)	13	39.4
None	9	27.3
Chemotherapy backbone		
Oxaliplatin-based (FOLFOX/CAPOX)	6	18.2
Irinotecan-based (FOLFIRI, Irinotecan)	10	30.3
FOLFOXIRI	3	9.1
Fluoropyrimidin mono	3	9.1
Encorafenib	10	30.3
Pembrolizumab	1	3.0

**Table 4:** Efficacy of first-line therapy

	All patients % (*N*)	Triplet (*N* = 12) % (*N*)	No triplet % (*N*)	moABX (*N* = 31) % (*N*)	No moABX (*N* = 20) % (*N*)
ORR (*N* = 39)	33.3 (13)	72.7% (8)	17.9% (5)	42.3% (11)	15.4% (2)
Odds ratio (95% CI)		OR = 12.267 (2.375–63.360) p* = 0.002		OR = 4.033 (0.740–21.983) p* = 0.151	
DCR	59.0 (23)	81.8% (9)	50% (14)	73.1% (19)	30.8% (4)
Odds ratio (95% CI)		OR = 4.500 (0.821–24.679) p* = 0.086		OR = 6.107 (1.415–26.356) p* = 0.017	
Median PFS (months) (*N* = 43) (95% CI)	8.4 (3.8–13.0)	13.0 (12.9–13.1)	4.3 (2.3–6.3)	10.5 (8.0–13.1)	3.1 (1.5–4.8)
Hazard ratio (95% CI)		*p*^#^ = 0.018 HR = 0.325 (0.121–0.873); * p* = 0.026		*p*^#^ = 0.063 HR = 0.474 (0.210–1.068); * p* = 0.072	
Median OS (months) (*N* = 51) (95% CI)	17.6 (12.6–22.7)	22.1 (14.7–29.4)	15.0 (9.9–20.1)	24.8 (19.3–30.3)	9.7 (3.4–16.0)
Hazard ratio (95% CI)		*p*^#^ = 0.404 HR = 0.730 (0.347–1.534); * p* = 0.406		*p*^#^ = 0.040 HR = 0.523 (0.279–0.981); * p* = 0.043	

*Two sided Fisher’s exact test; OR = odds ratio

^#^Log rank test; HR: hazard ratio

**Fig. 3:**
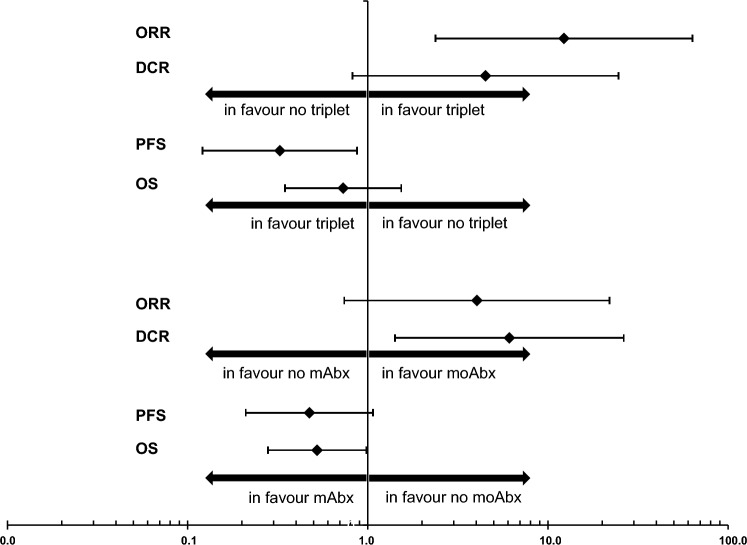
Forrest plot for odds ratios for overall response rate (ORR) and disease control rate (DCR) and hazard ratios for progression-free (PFS) and overall survival (OS) for patients which received a triplet chemotherapy in first line and a monoclonal antibody (mAbx), respectively

An intensive first-line chemotherapy with the triplet FOLFOXIRI was administered to *n* = 12 patients (Table 4). The ORR was significantly higher in patients treated with triplet chemotherapy (72.7%) than in those treated with other chemotherapy backbones (17.9%, *p* = 0.002). In line, the median PFS was significantly longer in patients receiving triplet chemotherapy (13.0 months vs 4.3 months; *p* = 0.018). However, this higher efficacy of FOLFOXIRI in first line did not translate into higher OS (*p* = 0.404) (Table 4 and Fig. 3). The addition of a monoclonal antibody to the palliative first-line chemotherapy backbone was observed for 31 patients (21 bevacizumab and 10 cetuximab or panitumumab). The use of an antibody was associated with a higher chance to achieve a disease control (73.1% vs. 30.8%, *p* = 0.017) (Table 4). The median PFS was numerical longer with the use of a monoclonal antibody (10.5 months vs 3.1 months). However, this difference was not statistically significant (*p* = 0.063). Interestingly, patients receiving an antibody had an unexpectedly high median OS of 24.8 months compared to patients without antibody treatment (9.7 months; *p* = 0.040) (Fig. 3). Surprisingly, this effect was independent of the class of monoclonal antibody: Patients treated with bevacizumab in first line had a median OS of 23.1 months (95% CI 13.5–32.7), and patients who received an EGFR antibody had a median OS of 26.4 months (95% CI 19.6–33.2) (*p* = 0.533). In multivariate Cox regression analyses with other known prognostic factors including age, primary tumour sidedness, sex and time of metastases (synchronous vs metachronous), we identified the use of a triplet chemotherapy as independent prognostic factor for PFS and the addition of a monoclonal antibody to first-line chemotherapy as independent prognostic factor for OS (suppl. Figure 3).

### Palliative second-line therapy

Upon disease progression on or after first-line therapy, 33 patients (64.7%) received a palliative second-line therapy. As encorafenib/cetuximab received EMA approval only in late May 2020, most patients were treated with an irinotecan- or oxaliplatin-based doublet chemotherapy in combination with an anti-angiogenic agent (bevacizumab, aflibercept or ramucirumab). However, 10 patients in our population already received a targeted therapy with the BRAF inhibitor encorafenib in combination with the EGFR antibody cetuximab as second line (Fig. 1 and suppl. table 2). The ORR to second line was only moderate with 8.7%, the DCR was 60.9% (suppl. table 3). The median PFS was 4.0 months (95% CI 3.8–4.3), and the median OS from start of palliative second line was 10.7 months (95% CI 6.3–15.0) (suppl. Figure 4A and B). The DCR upon encorafenib in combination with cetuximab was numerically higher with 87.5% compared to a classical cytotoxic chemotherapy (46.7%) (Table 4B). This difference did not reach statistical significance (*p* = 0.086) given the low number of patients. Interestingly, median PFS was comparable between patients receiving encorafenib or chemotherapy (4.1 vs 4.0 months; *p* = 0.697) (Table 5), while the median OS from start of the encorafenib-based therapy was almost twice as long as the median OS upon a cytotoxic chemotherapy (13.4 months vs 6.8 months; *p* = 0.329).

**Table 5:** Efficacy of second line with encorafenib and cetuximab

	Second line encorafenib (*N* = 10) % (*N*)	Second line w/o encorafenib (*N* = 23) % (*N*)
ORR to second-line therapy (*N* = 23)	0% (0)	13.3% (2)
	p* = 0.526 OR = 0.867 (95% CI 0.711–1.057)	
DCR to second-line therapy (*N* = 23)	87.5% (7)	46.7% (7)
	p* = 0.086 OR = 8.000 (95% CI 0.780–82.052)	
Median PFS (*N* = 21) (95% CI)	4.1 months (1.4–6.9)	4.0 months (2.7–5.3)
	*p*^#^ = 0.697 HR = 0.810 (0.280–2.344); * p* = 0.698	
Median OS from second line (*N* = 33) (95% CI)	13.4 months (9.7–17.1)	6.8 months (2.7–11.0)
	*p*^#^ = 0.329 HR = 0.625 (0.275–1.548); * p* = 0.332	

*Two sided Fisher´s exact test; OR = odds ratio

^#^Log rank test; HR: hazard ratio

In addition to the 10 patients receiving encorafenib/cetuximab as second-line treatment, 7 patients received a BRAF inhibitor-based therapy (encorafenib, dabrafenib or vemurafenib) beyond second line, and one patient was treated with encorafenib and cetuximab in the first-line setting (suppl. table 2 and 4A). Summing up, a total of 18 patients (35.3%) of our cohort received a BRAF inhibitor during their clinical course. The median OS of these patients from start of palliative treatment was highly promising with 25.1 months (95% CI 15.1–35.1) compared to only 13.1 months (95% CI 4.9–21.3) in those patients, who received cytotoxic chemotherapy only (*p* = 0.196; HR 0.656; 95% CI 0.344–1.249; *p* = 0.199) (Suppl. Table 4 and suppl. Figure 5). To exclude potential confounding factors, we compared the baseline characteristics of both patient populations. We found no difference regarding age, primary tumour location, time of metastases (synchronous vs metachronous), resection of primary tumour or received adjuvant chemotherapy between patients, which were treated with or without a BRAF inhibitor (Suppl. Table 5). The decisive factor for receiving BRAF-targeted treatment was an initial diagnosis in the years since 2010, which allowed patient inclusion in clinical trials or administration of targeted treatment as an approved drug.

## Discussion

Here, we report the clinical outcome of unselected patients with *BRAF*^*V600E*^-mutant mCRC, treated with palliative systemic therapy at a large comprehensive cancer centre. In contrast to prospective clinical trials in mCRC, we included patients irrespective of age, EOCG performance status, laboratory abnormalities or comorbidities in this retrospective analysis. The aim of this study was to identify the best treatment strategies for this poor prognostic patient population. As previously reported, patient characteristics were enriched with female sex, right-sided primary tumour, higher TNM stage at diagnosis and poor grading (G3) (Roth et al. 2010; Yamauchi et al. 2012; Clancy et al. 2013; Sinicrope et al. 2015; Wang et al. 2019). Most patients received a palliative doublet chemotherapy with FOLFOX or FOLFIRI in combination with bevacizumab as first line. Only a quarter (23.5%) of our real-world patients qualified for the intensive triplet FOLFOXIRI chemotherapy plus bevacizumab and were treated according to the current guidelines. These findings underline the challenges posed by the actual guidelines in a real-world patient population. However, those patients who received FOLFOXIRI had a median PFS of 13.0 months, which was comparable to the median PFS reported in the pivotal TRIBE study and significantly higher than in those patients who received a different first-line therapy (Cremolini et al. 2015). Furthermore, the ORR of 72.7% achieved with FOLFOXIRI was comparable to the control arm with FOLFOXIRI plus bevacizumab in the recently presented FIRE4.5 study (Stintzing et al. 2021). The median OS of 22.1 months in those FOLFOXIRI-treated patients was encouraging but it was not significantly higher compared to patients, receiving a different first-line therapy. This is in line with the recently published meta-analyses of 5 prospective randomized clinical trials in patients with mCRC, which investigated the additional benefit of the triplet chemotherapy with FOLFOXIRI plus bevacizumab versus a doublet with FOLFOX or FOLFIRI plus bevacizumab (Cremolini et al. 2020). In the subgroup of patients with *BRAF*^*V600E*−^mutant mCRC, the median OS was identical in patients who received a triplet or doublet first-line chemotherapy (13.6 vs. 14.5 months). Thus, the optimal first-line therapy in this poor prognostic patient population remains elusive. A doublet chemotherapy seems to be an appropriate alternative to FOLFOXIRI, in particular since the majority of patients in a real-world setting do not qualify for an intensive triplet chemotherapy. However, we recommend the addition of bevacizumab to the first-line chemotherapy backbone based on the results we observed in our patient population. Patients who received a monoclonal antibody had a higher ORR, median PFS and median OS compared to patients treated with chemotherapy only.

Interestingly, the median OS of 17.6 months in our unselected patient population was markedly higher than in the reported pooled analysis of Cremolini et al. and comparable to the recently reported data from the control arm with FOLFOXIRI plus bevacizumab of the FIRE4.5 trial (17.1 months) (Cremolini et al. 2020; Stintzing et al. 2021). This unexpectedly high median OS could be explained by the high number of patients, who had access to targeted therapies after progression following first-line therapy at our centre within clinical trials, as off-label use or later after the approval of encorafenib and cetuximab. In total, one third of our patients (18 out of 51) received a BRAF inhibitor in combination with an EGFR antibody or a different second targeted agent during the course of therapy. The median PFS in patients who received a BRAF inhibitor was 4.1 months and thus comparable to the median PFS observed in the pivotal BEACON trial with encorafenib and cetuximab (4.3 months) (Kopetz et al. 2019). Interestingly, the median PFS of 4.0 months in those patients receiving a standard chemotherapy in second-line in our cohort was comparable to the median PFS with encorafenib and cetuximab and markedly higher than the reported PFS of the control arm with cetuximab and irinotecan or FOLFIRI in the BEACON trial. This could be explained by the fact that only 3 patients in our cohort received an EGFR antibody in combination with cytotoxic chemotherapy and most patients were rather treated with a chemotherapy doublet in combination with an anti-angiogenic agent (bevacizumab or aflibercept) in second-line. A retrospective post-hoc molecular subgroup analyses of patients with *BRAF*^*V600E*^-mutated mCRC treated in the RAISE study, which evaluated the addition of the anti-angiogenic antibody ramucirumab to FOLFIRI in second line, also showed a median PFS of 5.7 months with a median OS of 9.0 months (Yoshino et al. 2019). Thus, cytotoxic chemotherapy should be rather combined with an anti-angiogenic agent rather than with an EGFR antibody in *BRAF*^*V600E*^-mutant mCRC patients. This was recently confirmed by the FIRE4.5 study which showed a superiority of bevacizumab versus cetuximab in first-line therapy with FOLFOXIRI.

Notably, in our cohort patients who received a BRAF inhibitor in combination with an EGFR antibody or a different targeted second agent had an extraordinarily high median OS of 25.1 months from start of their palliative treatment compared to only 13.1 months in those patients who received cytotoxic chemotherapy only. Thus, this treatment strategy should implicitly be integrated in the treatment algorithm of patients with *BRAF*^*V600E*^-mutant mCRC.

## Conclusion

In conclusion, only a small number of patients with *BRAF*^*V600E*^-mutant mCRC in a real-world setting qualify for the guideline recommended intensive first-line chemotherapy with FOLFOXIRI and are rather treated with a doublet chemotherapy in combination with bevacizumab. Even though the PFS in first line is prolonged with the triplet chemotherapy compared to other less intensive protocols, it has no significant impact on OS and a doublet chemotherapy with FOLFOX or FOLFIRI in combination with bevacizumab is an appropriate alternative. After progress to cytotoxic chemotherapy, the integration of a BRAF inhibitor in combination with an EGFR antibody into the treatment strategy in a real-world setting is feasible and highly recommended. Encouraging median OS times could be achieved in a real-world setting if patients have early access to these molecular targeted therapies.

## Supplementary Information

Below is the link to the electronic supplementary material.

Supplementary file1 (DOCX 186 KB)

## Data Availability

The authors confirm that the data supporting the presented results of this study are available within in the article and its supplemeentary material. Raw data that support the findings are available from the corresponding author, upon reasonable request.
