# Supplementary material for: Impact of encorafenib on survival of patients with BRAFV600E-mutant metastatic colorectal cancer in a real-world setting
Source: J Cancer Res Clin Oncol. 2023 Jul 19;149(14):12903–12. doi: 10.1007/s00432-023-05141-y (PMC10587317; doi:10.1007/s00432-023-05141-y)
Supplement: Supplementary file 1 — Supplementary file1 (DOCX 186 KB) [file 432_2023_5141_MOESM1_ESM.docx]

**Suppl. Figure 1: Consort-Diagram**

BRAF-population

N=81

N=63

23 pts. w/o clinical data

treated-population

N=58

2 pts. with atypical BRAF mutations

BRAFV600E-population

N=56

5 pts. w/o relapse/metastases

study-population

N=51

**Suppl. Table 1: tumor characteristics**

|  | **N** | **%** |
| --- | --- | --- |
| Localization of primary tumor  ileocoecal  ileocoecal+ c. ascendens  coecum  c. ascendens+rectal  c. ascendens  flexura coli dextra  c. transversal  c. descendens  sigmoideum  rectum  unknown primary (NEC) | 1  1  6  1  23  1  4  1  4  7  1 | 2.0  2.0  11.8  2.0  45.1  2.0  7.8  2.0  7.8  13.7  2.0 |
| Histology  adenocarcinoma  neuroendocrine carcinoma  mixed adenoneuroendocrine (MANEC) | 49  1  1 | 96.1  2.0  2.0 |
| Grading  G1  G2  G3  Gx/n.d. | 1  20  19  11 | 2.0  39.2  37.3  21.6 |
| T-stage (initial)  T1  T2  T3  T4  Tx/n.d. | 0  2  25  12  12 | 0  3.9  49.0  23.5  23.5 |
| N-stage (initial)  N0  N1  N2  Nx | 5  12  21  13 | 9.8  32.5  41.2  25.5 |
| M-stage (initial)  M0  M1  Mx/n.d | 14  37  0 | 27.5  72.5  0 |
| Stage UICC (initial)  0  I  II  III  IV | 0  0  2  13  37 | 0  0  2.0  25.5  72.5 |
| Site of metastases (synchronous/metachronous)  liver  lung  peritoneal  lymphnodes  other (bone, skin, ovar) | 28  10  12  3  6 |  |

**Suppl. Figure 2: Relapse free survival (RFS) (resected patients without synchronous metastases, N=14)**


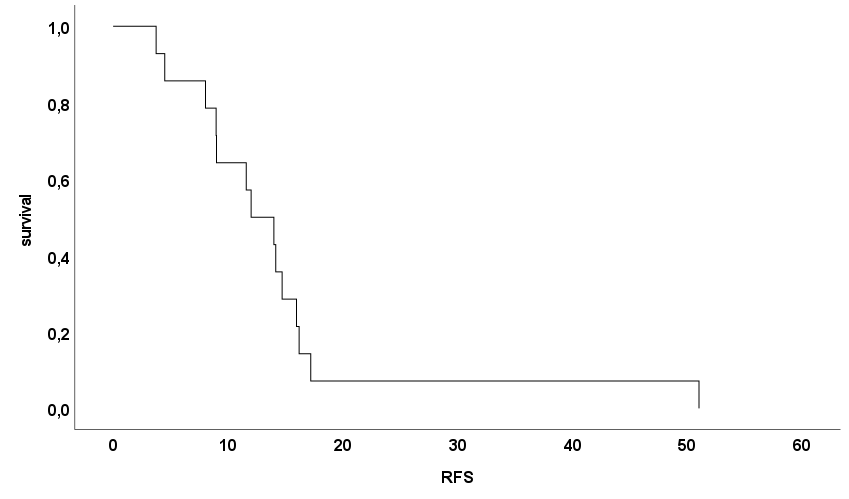


| **RFS** | **Events** | **Censored** | **median** | **95%CI** |
| --- | --- | --- | --- | --- |
| months | 14 | 0 | 12.0 | 7.6-16.4 |

**Suppl. Table 2: Palliative chemotherapy regimen**

|  | **N** | **%** |
| --- | --- | --- |
| 1^st^ line chemotherapy (N=51)  BSC  FOLFOX/CAPOX  FOLFIRI  Irinotecan mono  FOLFOX+Cetuximab or Panitumumab  FOLFIRI+Bevacizumab  5-FU/FA+Bevacizumab  FOLFOX+Bevacizumab  5-FU/FA  FOLFOXIRI+Bevacizumab  Cisplatin/Etoposid  FOLFIRI+Cetuximab or Panitumumab  Encorafenib+Cetuximab  FOLFOXIRI+Panitumumab  n.d. | 3  8  3  1  6  4  1  5  2  11  2  2  1  1  1 | 5.9  15.7  5.9  2.0  11.8  7.8  2.0  9.8  3.9  21.9  3.9  3.9  2.0  2.0  2.0 |
| 2^nd^ line Chemotherapy (N=33)  FOLFOX/CAPOX  FOLFIRI  Pembrolizumab  Trifluridin/Tipiracil  Encorafenib+Cetuximab+PI3K Inhibitor  Irinotecan+Cetuximab  FOLFIRI+Bevacizumab  5-FU/FA+Bevacizumab  FOLFOX+-Bevacizumab  Irinotecan+Aflibercept  FOLFOXIRI+Bevacizumab  FOLFOX+Panitumumab  Encorafenib+Cetuximab  FOLFOXIRI+Cetuximab  Encorafenib+Binimetinib+Cetuximab  FOLFIRI+Aflibercept | 1  5  1  2  1  1  2  1  4  1  2  1  6  1  3  1 | 3.0  15.2  3.0  6.1  3.0  3.0  6.1  3.0  12.1  3.0  6.1  3.0  18.2  3.0  9.1  3.0 |
| 3^rd^ line Chemotherapy (N=23)  FOLFOX/CAPOX  Irinotecan mono  Cetuximab  Panitumumab  Regorafenib  Pembrolizumab  Trifluridin/Tipiracil  Irinotecan+Cetuximab  FOFIRI+Bevacizumab  5-FU+Bevacizumab  FOLFOX+Bevacizumab  FOLFOXIRI+Bevacizumab  FOLFIRI+Panitumumab  Encorafenib+PI3K Inhibitor+Cetuximab  Encorafenib+Cetuximab  Encorafenib+Binimetinib+Cetuximab  Darbrafenib+ERK1/2 inhibitor | 2  1  2  2  1  1  1  3  1  1  1  1  1  2  1  1  1 | 8.7  4.3  8.7  8.7  4.3  4.3  4.3  13.0  4.3  4.3  4.3  4.3  4.3  8.7  4.3  4.3  4.3 |
| 4^th^ ine Chemotherapy (N=7)  Cetuximab  Pembrolizumab  Irinotecan+Cetuximab  5-FU+Bevacizumab  Encorafenib+Cetuximab  Encorafenib+Binimetinib+Cetuximab | 1  2  1  1  1  1 | 14.3  28.6  14.3  14.3  14.3  14.3 |
| 5^th^ line Chemotherapy (N=3)  Irinotecan  FOLFOX+Bevacizumab  Vemurafenib+Cetuximab | 1  1  1 | 33.3  33.3  33.3 |

**Suppl. Figure 3: Multivariate analyses of 1^st^ line therapy**

| **Progression-Free Survival** |
| --- |
| **Prognostic factor** |
| Triplet |
| mAbx received |
| Primary tumor side |
| Metachronous metastases |
| Age (Median) |
| Sex |

|  |  |  |
| --- | --- | --- |
| **HR** | **CI** | **p** |
| 0.311 | (0.102-0.952) | 0.041 |
| 00.377 | (0.117-1.210) | 0.101 |
| 1.568 | (0.637-3.860) | 0.328 |
| 0.549 | (0.173-1.747) | 0.310 |
| 1.210 | (0.488-2.998) | 0.681 |
| 2.721 | (1.121-6.602) | 0.027 |

0.1

10.0

1.0

| **Overall Survival** |
| --- |
| **Prognostic factor** |
| Triplet |
| mAbx received |
| Primary tumor side |
| Metachronous metastases |
| Age (Median) |
| Sex |

|  |  |  |
| --- | --- | --- |
| **HR** | **CI** | **p** |
| 0.584 | (0.221-1.543) | 0.278 |
| 0.318 | (0.130-0.780) | 0.012 |
| 0.757 | (0.390-1.468) | 0.410 |
| 0.499 | (0.211-1.181) | 0.114 |
| 1.516 | (0.755-3.043) | 0.242 |
| 2.956 | (1.415-6.176) | 0.004 |

0.1

10.0

1.0

mAbx: monoclonal antibody


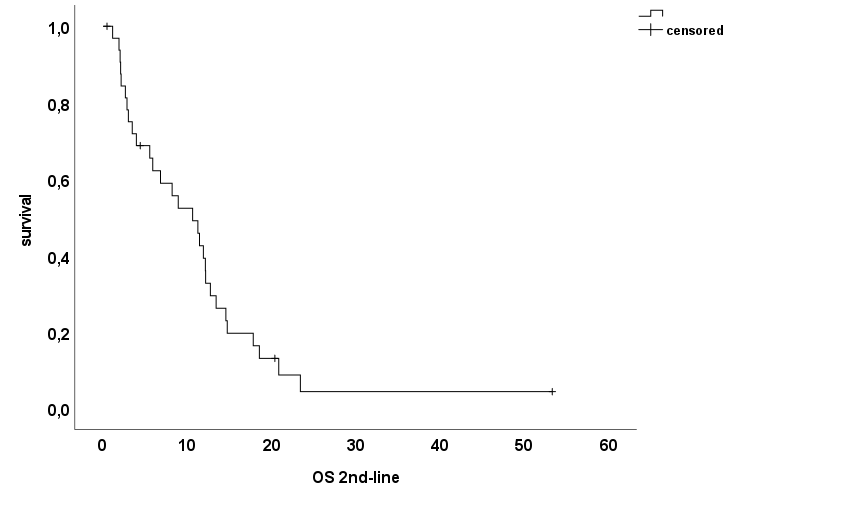
**Suppl. Figure 4A: Overall survival to second line therapy**

| **OS 2^nd^ line** | **N** | **Censored/death** | **median** | **95%CI** |
| --- | --- | --- | --- | --- |
| months | 33 | 4/29 | 10.65 | 6.29-15.00 |

**Suppl. Figure 4B: progression free survival (PFS) to second line therapy**


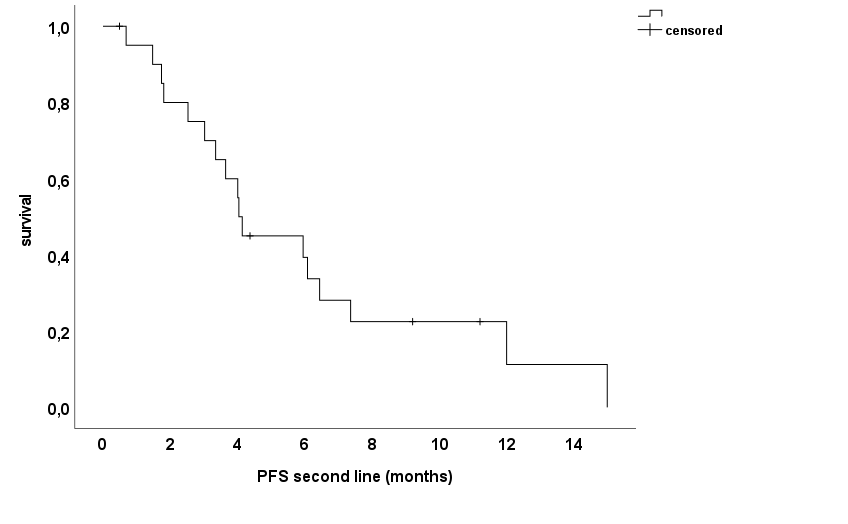


| **PFS 2^nd^ line** | **N** | **Censored/PD** | **median** | **95%CI** |
| --- | --- | --- | --- | --- |
| months | 21 | 4/17 | 4.04 | 3.75-4.33 |

**Suppl. Table 3 efficacy of 2^nd^ line therapy**

|  | **N** | **%** |
| --- | --- | --- |
| Best ORR to 1^st^ line therapy (N=23)  CR  PR  SD  PD | 0  2  12  9 | 0  8.7  52.2  39.1 |
| ORR  Yes  No | 2  21 | 8.7  91.3 |
| DCR  Yes  No | 14  9 | 60.9  39.1 |
| Median PFS (N=21) (95% CI) | 4.0 months (3.8-4.3) | |
| Median OS (N=33) (95%CI) | 10.7 months (6.3-15.0) | |

**Suppl. Table 4A BRAF inhibitor therapy**

|  | **N** | **%** | **95% CI** |
| --- | --- | --- | --- |
| BRAF inhibitor received  Yes  No | 18  33 | 35.3  64.7 | 21.6-49.0  51.0-78.4 |

**Suppl. Table 4B Overall survival of patients with BRAF inhibitor therapy**

| **OSpall** | **N** | **Censored/death** | **median** | **95%CI** | **Log rank** | **HR (95%CI)** |
| --- | --- | --- | --- | --- | --- | --- |
| BRAF inhibitor received | 18 | 4/14 | 25.1 | 15.1-35.1 | p=0.196 | 0.656 (0.344-1.249), p=0.199 |
| No BRAF inhibitor received | 33 | 3/30 | 13.1 | 4.9-21.3 |  |  |

**Suppl. Figure 5: Overall survival from start of palliative treatment in patients which received a BRAF inhibitor**


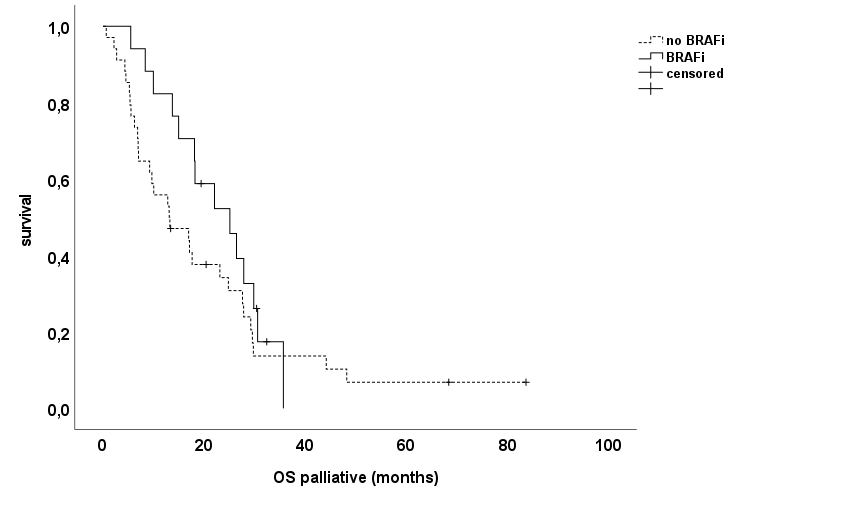


| **OD months** | **N** | **Censored/death** | **median** | **95%CI** |
| --- | --- | --- | --- | --- |
| BRAFi | 18 | 4/14 | 25.1 | 15.1-35.1 |
| no BRAFi | 33 | 3/30 | 13.1 | 4.9-21.3 |

**Suppl. Table 5 patients´characteristics of patients which were treated with or without a BRAF inhibitor**

|  | **no BRAF inhibitor (N=33)** | | **BRAF inhibitor (N=18)** | | **p-value** |
| --- | --- | --- | --- | --- | --- |
|  | **N** | **%** | **N** | **%** |  |
| Gender  male  female | 15  18 | 45.5  54.5 | 8  10 | 44.4  55.6 | 0.945* |
| age at diagnosis  median (range) | 63.7 years (40.5-88.4) | | 57.2 years (42.8-77.9) | | 0.306^#^ |
| age at metastases/relapse  median (range) | 63.7 years (41.4-88.4) | | 57.8 years (42.8-79.3) | | 0.287^#^ |
| Tumor localization  Left sided  Right sided  Unknown/both | 9  23  1 | 27.3  69.7  3.0 | 3  14  1 | 16.7  77.8  5.6 | 0.653* |
| M-stage  synchronous M1  metachronous M1 | 23  10 | 69.7  30.3 | 14  4 | 77.8  22.2 | 0.537* |
| Resection of primary tumor  Yes  No | 26  7 | 78.8  21.2 | 12  6 | 66.7  33.3 | 0.343* |
| Adjuvant CTX (only resected with metachronous metastases; N=14)  Yes  No | 6  4 | 60.0  40.0 | 3  1 | 75.0  25.0 | 0.597* |

*Chi-Square; ^#^t-test
